# The Impact of High Burden Idiopathic Premature Ventricular Contractions in Pediatric versus Adult Populations. A Retrospective Cohort Study

**DOI:** 10.1007/s00246-025-03784-x

**Published:** 2025-02-02

**Authors:** Lamyaa Elsayed Allam, Mervat Aboulmaaty Nabih, Mohamed Basyouni Helal, Abdallah R. Allam, Ahmed Nabil Ali

**Affiliations:** 1 Cardiology Department, Faculty of Medicine, Ain Shams University, 48 Mohammed Elnadi Street, 6Thzonezone, Nasr CityCairo, P0 11371 Egypt; 2 Faculty of Medicine, Menoufia University, Menoufia, Egypt

**Keywords:** Idiopathic PVC, Outflow tract PVC, ECG, RF ablation, PVC-induced cardiomyopathy, High-burden PVC

## Abstract

Idiopathic premature ventricular contractions (PVCs) can occur in up to 40% of children with structurally normal hearts. The study aims to assess the impact of high-burden idiopathic PVCs on children in comparison with adults in terms of symptoms, distribution of origin, management strategies, and safety considerations. This is a single-center retrospective cohort study that included patients with frequent PVCs (> 10% burden in Holter monitoring) and categorized by age into adult group (≥ 18 years) and pediatric group. Data encompassed demographic profiles, clinical symptoms, echocardiographic findings, electrocardiographic analyses, and one-year follow-up management. A total of 224 PVC cases were collected during a 2-year study (120 children and 104 adults). Syncope occurred significantly more in children than adults (15.8% vs. 0.9%, *p* = 0.003). In children, the right ventricular outflow tract (RVOT) free wall was the predominant site (48 patients; 40%), while the septal RVOT was most common in adults (35 patients; 33.7%). Pediatric cases exhibited significantly higher PVCs originating from the outflow tract (92.5% vs. 69.2%; *p* = 0.001). Nevertheless, there were no significant differences between groups regarding the safety, acute, and one-year outcomes of RF ablation. Age disparities were evident in syncope incidence and PVC origin, with no variance in PVC-induced myopathy prevalence or the safety and outcomes of RF catheter ablation between children and adults.

## Introduction

Idiopathic premature ventricular contractions (PVCs) occur in the absence of any structural cardiac abnormality [1]. It is estimated that up to 40% of healthy children have PVCs when performing ambulatory 24-h Holter monitoring [2]. Depending on the screening type and duration, the prevalence of idiopathic PVCs is estimated to be between 10 and 30% in children without apparent structural heart disease [3]. With frequent ectopy (generally defined as ≥ 10 percent of beats in 24 h), there is a risk of developing ventricular dysfunction even in a normal heart, which has been proven in adults in several studies but has still not been studied well in children [4]. Although PVCs are generally considered a benign condition in the pediatric population, there is a small percentage in this age group with a greater risk of developing life-threatening forms of ventricular tachycardia (VT) or ventricular fibrillation (VF) [4].

Unfortunately, antiarrhythmic medications are not significantly effective in children in many cases [5]. As stated in the PACES/HRS expert consensus statement on the use of catheter ablation in children, ablation of frequent PVCs in children is strongly indicated in cases of left ventricular dysfunction caused by a single focused PVC if medical treatment failed or was intolerable [3]. Besides a reduced left ventricular ejection fraction, frequent PVCs may be highly symptomatic, leading to a significantly reduced quality of life, and radiofrequency (RF) catheter ablation is also useful in pediatric patients [6].

The available literature on the nature of idiopathic PVCs and the efficacy of RF catheter ablation in children compared to adults is limited to case reports and small single-center retrospective series. This study aims to compare the impact of high-burden idiopathic PVCs on children and adults regarding symptoms, distribution of origin, management strategies, and safety considerations.

## Methods

### Study Design and Setting

This is a retrospective cohort study that included patients referred to the cardiac electrophysiology unit at Ain Shams University Hospitals with symptomatic high-burden PVCs (> 10% of total beats per day) for electrophysiological study and RF catheter ablation. Those patients were referred from the outpatient clinic or other centers.

### Participants

The participants were included if they had up to two PVC morphologies, at least 10% ventricular ectopy burden on a 24–48 h Holter monitor, and a structurally normal heart. Exclusion criteria were the presence of structurally abnormal heart-like congenital heart disease (except for a patent foramen oval), history of surgical repair for congenital heart disease, cardiomyopathies (as hypertrophic or dilated), ‘burnt-out’ myocarditis, and coronary artery disease. Also, patients with cardiac tumors, electrical heart disease (including channelopathies such as long QT syndrome, Brugada syndrome, and catecholaminergic polymorphic VT), chest wall trauma, and systemic illnesses that could affect outcome (e.g.: HIV, cystic fibrosis) were excluded from this study. The patient population was divided into 2 groups according to their age: A pediatric group, which included all patients who were less than 18 years old (according to the recent definition by the WHO organization); and an adult group, including all other patients who were more than 18 years old.

### Clinical Assessment

A detailed assessment was done for each participant in each group, including history taking with special emphasis on symptoms experienced by the patient due to premature beats such as palpitations, chest pain, dyspnea, dizziness, or fainting episodes. A complete general and cardiac examination was done on all patients. A twelve-lead electrocardiogram (ECG) was obtained, aiming at proper localization of PVC origin, and detecting if there were different morphologies of PVC, indicating different foci of PVC initiation. A detailed algorithm done by Xiong et al. [7] was used to determine the site of origin of PVC. Echocardiography was done on all patients with an assessment of left ventricular systolic functions, dimensions, and volumes, determining any cases of PVC-induced cardiomyopathy in the form of dilatation of LV dimensions and volumes, and impairment of LV systolic functions. Ambulatory 24–48 h Holter monitoring was done to detect the burden of PVC per day (defined as the number of PVC as a percentage of the total beats per 24 h) and their distribution time throughout the day (whether they were more during the day or night), in addition to the determination of morphology consistency to exclude polymorphic PVCs. In patients with suspected PVC-induced myopathy, cardiac MRI was done to exclude other causes of impaired LV systolic function based on the ESC guidelines published in 2022 [8].

### Management Strategy

#### Pharmacological Therapy

We assessed the efficacy of antiarrhythmic drugs (AADs) to improve symptoms. The choice of AADs was randomly made either in the form of Class IC AADs, Class II beta-blockers, Class III (sotalol or amiodarone), or Class IV non-dihydropyridine calcium channel blockers (CCBs). A close monitoring of all patients was done to follow-up on their symptoms, and improvement in their quality of life, in addition to possible drug side effects and PVC burden, by Holter monitoring after 3 months. If PVC burden was > 10% of total beats or symptoms were still persistent, the patient was referred for an EP study and RF catheter ablation.

#### EP Study and RF Ablation

In patients with no improvement in the symptoms or the burden of PVCS by medical therapy or patients who developed PVC-induced cardiomyopathy, the decision was to perform an electrophysiology (EP) study with RF catheter ablation. AADs were discontinued for at least 5 half-lives before the procedure. All procedures were done under local anesthesia. Two venous accesses were used to introduce a 6 Fench (Fr) diagnostic quadripolar and a 6 Fr deflectable decapolar catheter. By utilizing three-dimensional electro-anatomical mapping (CARTO® system by Biosense Webster, USA), local activation and substrate maps were done using the mapping catheter to determine the desired target of ablation at the earliest activation potential in the map. Detailed mapping with the ablation catheter was used to target the earliest site of activation of PVC, with a cut-off value of − 30 ms (ms) pre-QRS in RVOT and − 25 ms in LVOT and non-outflow tract PVC [9, 10]. RF ablation was performed between 30–40 Watts with a temperature target between 45–55 °C for about 120 s. At the end of the procedure, the lack of any PVCs with the same morphology within about 30 min after ablation was considered acute success.

### Follow Up

After the procedure, routine follow-up visits were held in the outpatient clinic at 1, 3, 6, and 12 months. Throughout the follow-up, 24–48 Holter monitoring was performed regularly. Additional monitoring was carried out if symptoms returned or PVCs were discovered on an ECG. A reduction in PVC burden by more than 80% of the pre-procedural PVC load was considered an indicator of a successful ablation procedure after a one-year follow-up. [11, 12] Recurrence was defined as the reappearance of arrhythmias with the same features as the ablated arrhythmia during the follow-up period.

### Outcomes

A comparison was done between the pediatric group and adult group regarding baseline characteristics, symptoms, PVC localization by ECG, echocardiography data, and response to different management strategies, including outcomes and safety of RF catheter ablation.

### Ethical Consideration

Ethical approval for the study was obtained from the IRB committee of Ain-shams Faculty of Medicine, ensuring compliance with ethical standards and regulations governing human research. Written informed consent was obtained from all participants before the study’s commencement.

### Data Analysis

Data were analyzed using IBM SPSS Statistics for Windows version 28 (IBM Corp., Armonk, N.Y., USA). Frequencies and percentages were used to describe the categorical variables. For continuous variables, we tested the normality using the Shapiro–Wilk test. The median and interquartile range (IQR) were used to describe the non-parametric variables. The chi-square test and Fisher’s Exact test were used to assess the associations between the categorical variables. Furthermore, a paired-sample t-test was used to compare the mean Holter burden before and after the management in both groups. The Kruskal–Wallis test was used to compare the difference between the effectiveness of the drug classes in decreasing the Holter burden. A p-value less than 0.05 was considered statistically significant.

## Results

### Baseline Characteristics

A total of 224 PVC cases were collected during a 2-year study. 120 cases were children with a median age of 14, IQR = 6, and 65 (54.2%) were male. In contrast, 104 cases were adults with a median age of 43.5, IQR = 24, and 49 (47.1%) were male (Table 1).

**Table 1:** Comparison of baseline characteristics, PVC morphology on ECG, and prevalence of PVC-induced myopathy according to age groups

Variable	Children (*n* = 120)	Adults (*n* = 104)	*P*-value*
Age (years)	14 (6)	43.5 (24)	
Gender			
- Female	55 (45.8%)	55 (52.9%)	
- Male	65 (54.2%)	49 (47.1%)	
Weight (Kg)	58 (26)	75 (22)	
Symptoms			0.003*
- Palpitations	81 (67.5%)	84 (80.8%)	
- Chest pain	14 (11.7%)	11 (10.6%)	
- Dyspnea	6 (5%)	8 (7.7%)	
- Syncope	19 (15.8%)	1 (0.9%)	
- Dizziness	0 (0%)	0 (0%)	
- Cardiac arrest	0 (0%)	0 (0%)	
PVC burden percentage (%), mean ± SD	24.2 ± 8.3	26.4 ± 9.3	0.053
PVCs provisional localization			0.001*
- Outflow tract	111 (92.5%)	72 (69.2%)	
- Non-outflow tract	9 (7.5%)	32 (30.8%)	
PVCs precise localization (on ECG)			< 0.001*
- AMC	3 (2.5%)	9 (8.7%)	
- LCC	23 (19.2%)	14 (13.5%)	
- LV summit	2 (1.7%)	16 (15.4%)	
- Papillary muscle	1 (0.8%)	3 (2.9%)	
- Parahisian	0 (0%)	2 (1.9%)	
- RCC	10 (8.3%)	7 (6.7%)	
- RCC-LCC junction	15 (12.5%)	4 (3.8%)	
- RVOT-free wall	48 (40%)	12 (11.5%)	
- RVOT septal	15 (12.5%)	35 (33.7%)	
- Tricuspid annulus	3 (2.5%)	2 (1.9%)	
- Total	120 (100%)	104 (100%)	
BBB morphology			0.031*
- LBBB like	113 (94.2%)	89 (85.6%)	
- RBBB like	7 (5.8%)	15 (14.4%)	
Left Ventricular EF			0.149
- Normal EF	118 (98.3%)	98 (94.2%)	
- Impaired EF (< 50)	2 (1.7%)	6 (5.8%)	

### Symptoms

The analysis revealed varying clinical presentations of PVC between both groups. Among those who experienced symptoms, the sense of palpitations was the most commonly reported symptom, with 84 (80.8%) of adults and 81 (67.5%) of children. Despite that, only one adult patient suffered from syncope; in contrast, 19 children (15.8%) experienced it.

There was a significant difference in symptom prevalence between the two groups (*p* = 0.003). Post-hoc testing using Bonferroni adjustment showed that the percentage of syncope among children (15.8%), is significantly higher than that among adults (0.9%). No difference in other symptoms was observed between children and adults (Table 1).

In patients who experienced syncope, additional investigations were done to determine the cause of syncope, including both extended ECG monitoring and the Tilt table test. We found that 14 patients had type III (pure vasodepressor type) neurocardiogenic syncope, 3 patients had type I (mixed type) neurocardiogenic syncope, and only 2 patients had attacks of sustained ventricular tachycardia during syncope at attacks in the pediatric group, while in the adult group, the only patient who had syncope, had type III of neurocardiogenic syncope.

### PVC Morphology on ECG

According to the morphological characteristics of the PVCs on 12-lead electrocardiograms (ECG), 113 (94.2%) cases were LBBB-like in the pediatric group, whereas it was 89 (85.6%) in the adult group (P 0.03). PVCs originating from the outflow tract showed a significantly higher percentage in the pediatric group compared to the adult group (92.5 vs. 69.2%) (*p* = 0.001).

In children, the RVOT-free wall site was the most prevalent localization of PVCs 48 (40%), while in the adult group, the septal RVOT site exhibited a considerable number (35 patients, 33.7%).

**Fig. 1:**
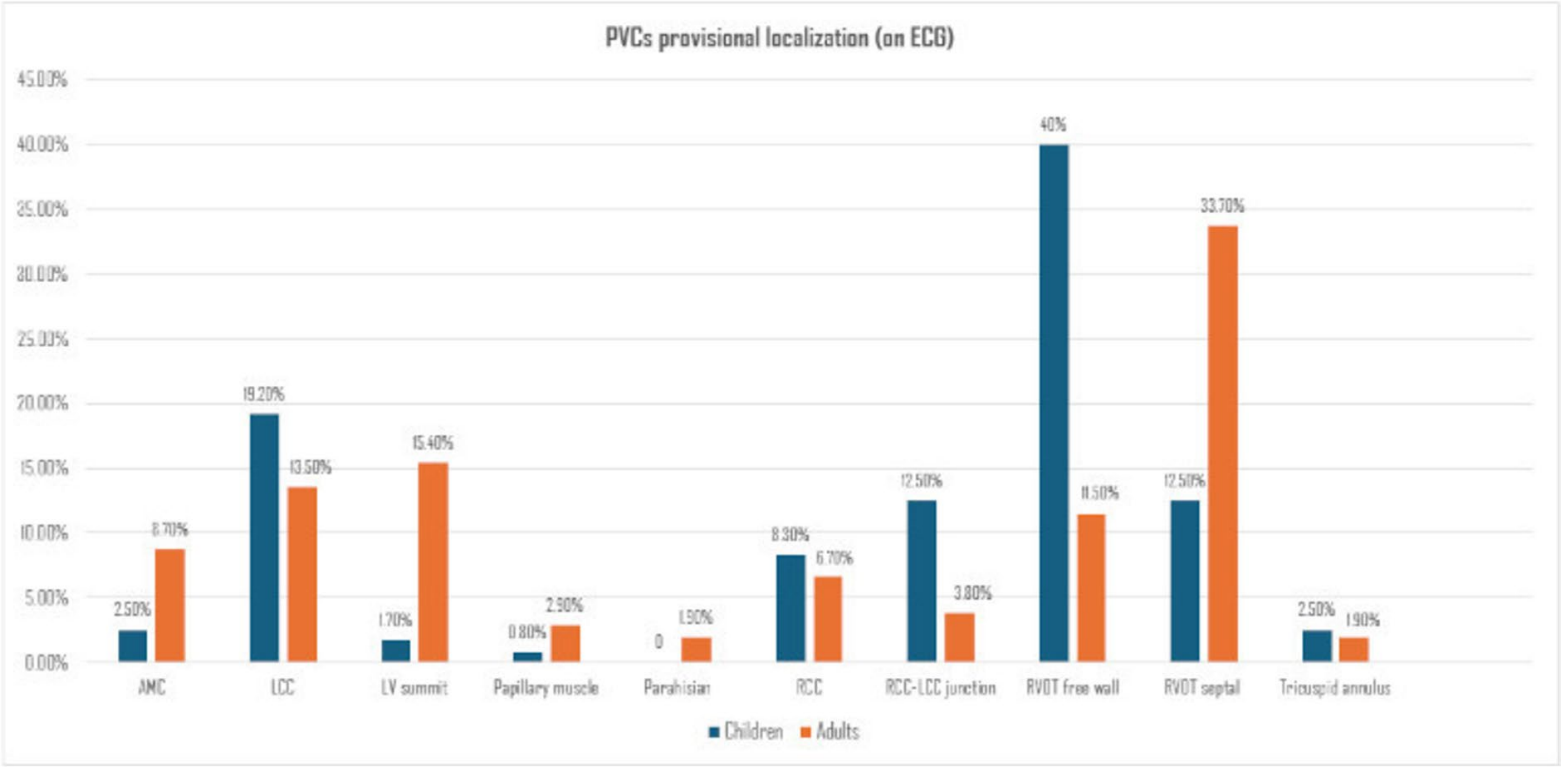
PVC provisional localization (on ECG) among children and adult patients

The Chi-square test suggested that there was a significant difference in the precise localizations of PVC between the two groups (*p* < 0.001). Post-hoc testing using Bonferroni adjustment revealed that the percentage of RCC-LCC junction and RVOT-free wall among the children group (12.5%, 40%) were significantly higher than the adult group (3.8%, and 11.5%), respectively. While the percentage of RVOT septal, LV summit, and aortic mitral continuity among adults (33.7%, 15.4%, 8.7%) is significantly higher than that of children (12.5%, 1.7%, 2.5%). There was no difference in the other PVCs’ precise localization between the two age groups (Table 1 and Fig. 1).

The age and weight are presented with the median (IQR).

PVC provisional localization, BBB morphology, and precise locations are presented with N (%).

*Chi-square test and Fisher’s exact test; significant *p*-value < 0.05.

*Kg* Kiliograms; *AMC* Aortomitral Continuity; *RCC* Right Coronary Cusp; *LCC* Left Coronary Cusp; *RVOT* Right Ventricular Outflow Tract; *LV summit* Left Ventricular Summit; *LBBB* Left Bundle Branch; *RBBB* Right Bundle Branch; *PVC* Premature Ventricular Contractions; *EF* Ejection Fraction.

### Baseline Echocardiographic Data

Regarding PVC-induced myopathy, there was no statistical difference between both groups regarding the prevalence of PVC-induced myopathy between both groups (Table 1).

In addition, there was no statistically significant association between PVC-induced myopathy and the localization of PVC in both age groups (Table 2).

**Table 2:** Relationship between PVC-induced myopathy and localization of PVC in both age groups

	Children		*P*-value*	Adults		*P*-value*
	Outflow tract PVC (N = 111)	Non-outflow tract PVC (N = 9)		Outflow tract PVC (N = 72)	Non-outflow tract PVC (N = 32)	
Normal LV EF	109 (92.4%)	9 (7.6%)	1	70 (71.4%)	28 (28.6%)	0.071
Impaired LV EF (< 50)	2 (100%)	0 (0%)		2 (33.3%)	4 (66.7%)	

*Fisher’s Exact Test; significant *p*-value < 0.05

LV EF, left ventricular ejection fraction; PVC, premature ventricular contractions

### Management Strategy

Regarding the management of PVCs, various strategies, including medical and ablation, were employed in both adult and pediatric cohorts.

### Pharmacological Therapy

For the pediatric patients, 106 patients received pharmacological therapy first due to the refusal of their parents to undergo RF ablation. Pharmacological therapy was effective in improving symptoms and achieving a reduction in the PVC burden in 59 out of 106 patients (55.6%), while the other 47 patients were transferred for ablation. However, 14 (11.67%) cases underwent ablation from the start.

In contrast, none of the adult cohorts underwent medical treatment because of the preference of the patients to not receive any medications or previous failed medical therapy before transfer to our center, besides the presence of medical insurance coverage for these procedures in our center.

In pediatric patients who received only medical therapy, all evaluated drug classes showed a reduction in the Holter burden. However, no significant difference was observed between the five drug classes in their effectiveness at decreasing the Holter burden (p = 0.306). These drug classes included beta-blockers, non-dihydropyridine calcium channel blockers (NDH-CCB), Class IC antiarrhythmic drugs, and sotalol.

### Interventional Therapy (EP Study and RF Ablation) in Both Groups

Regarding the ablation procedure, it was done in almost half of the pediatric patients (61 out of 120, 50.1%) and in all patients in the adult group.

An aortogram (injecting at the level of the aortic cups) was performed in only 8 cases with left outflow tract PVCs in the pediatric group due to their small body size. This was done to delineate the aortic cusp anatomy and coronaries’ ostia).

For the pediatric group, 57 out of 61 cases who underwent ablation achieved acute success, resulting in a success rate of 93.4%. Most children (58 cases, 95.1%) did not experience any complications, with only 3 cases (4.9%) having puncture hematoma.

In the adult group, 89 out of 104 cases who underwent ablation achieved acute success, yielding a success rate of 85.6%. The majority of adults (102 cases, 98%) did not experience complications, with only 2 cases (2%) reporting puncture hematoma (Table 3).

**Table 3:** Acute Success Rate, complications, and Site of PVCs during Ablations in both groups

	Children (*N* = 61)	Adults (*N* = 104)	*P*-value*
Acute success	57 (93.4%)	89 (85.6%)	
Complications			
– Puncture hematoma	3 (4.9%)	2 (2%)	
– None	58 (95.1%)	102 (98%)	
PVC’s precise localization (on ablation)			< 0.001*
- AMC	1 (1.6%)	8 (7.7%)	
- Anterior papillary muscle	0 (0%)	3 (2.9%)	
- Anterior tricuspid annulus	1 (1.6%)	0 (0%)	
- Anterolateral mitral annulus	0 (0%)	1 (1.0%)	
- Lateral tricuspid annulus	0 (0%)	1 (1.0%)	
- LCC	15 (24.7%)	14 (13.4%)	
- LV summit	0 (0%)	16 (15.4%)	
- Parahisian	0 (0%)	2 (2.0%)	
- RCC	6 (9.8%)	7 (6.7%)	
- RCC-LCC junction	5 (8.2%)	4 (3.8%)	
- RVOT-free wall	27 (44.3%)	13 (12.5%)	
- RVOT septal	1 (1.6%)	22 (21.1%)	
- Subpulmonic	5 (8.2%)	10 (9.6%)	
- Supra pulmonic	0 (0%)	2 (1.9%)	
- Tricuspid annulus	0 (0%)	1 (1.0%)	
- Total	61 (100%)	104 (100%)	

### Sites of the Successfully Ablated PVCs

**Fig. 2:**
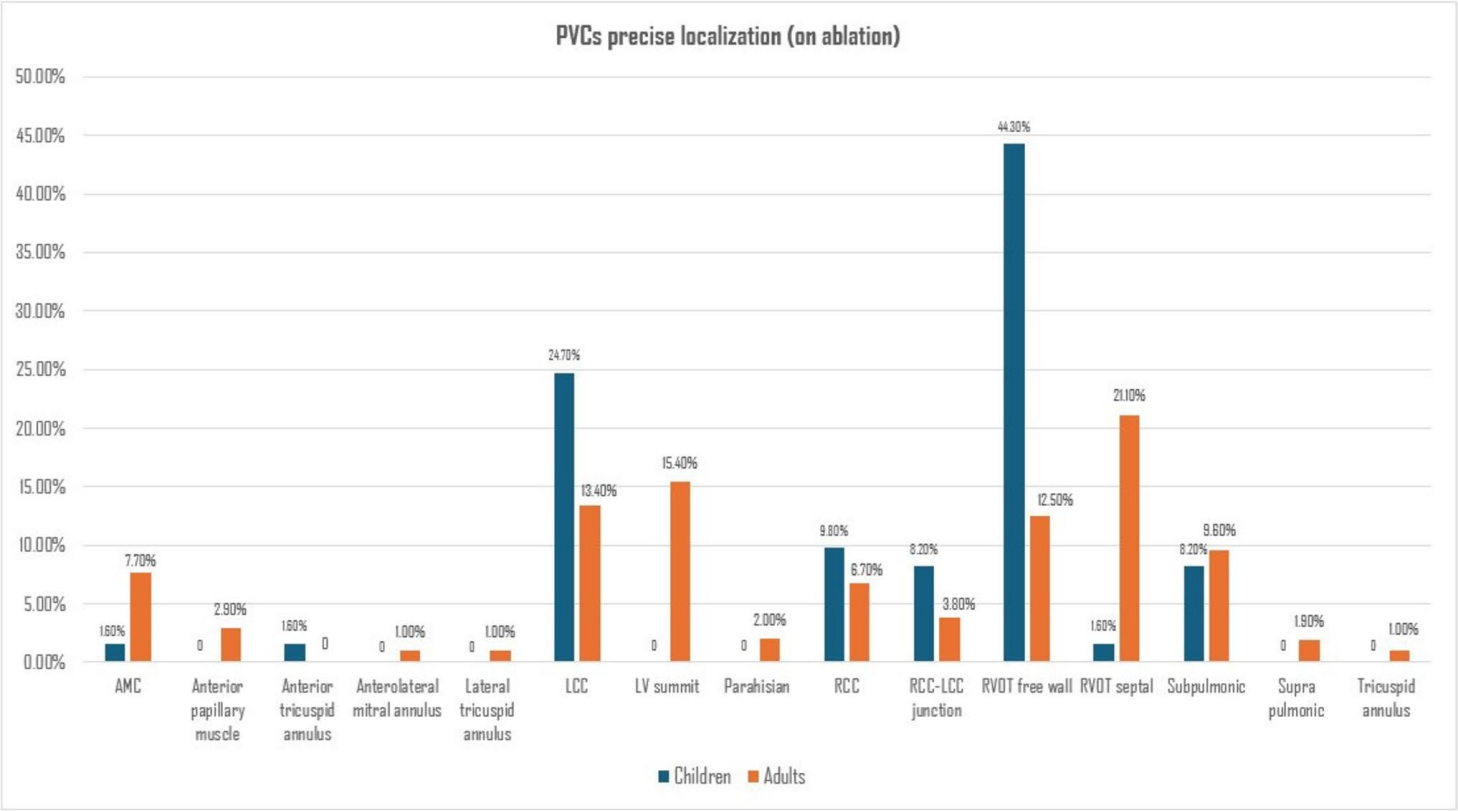
Successful ablated sites of PVCs among children and adult patients

The Chi-square test suggested that there was a significant difference between the percentage of sites of PVCs between the two groups (*p* < 0.001). Post-hoc testing using Bonferroni adjustment revealed that the percentage of RVOT-free walls among the children group (27 cases, 44%) was significantly higher than the adult group (13 cases, 12.5%). While the percentage of RVOT septal, LV summit, and aortic mitral continuity among adults (21%, 15.4%, and 8%) were significantly higher than children (1.6%, 0%, and 1.6%), respectively. There was no difference in the other PVCs’ precise localization between the two age groups (Table 3 and Fig. 2).

*RCC* Right Coronary Cusp; *LCC* Left Coronary Cusp; *RVOT* Right Ventricular Outflow Tract; *LV summit* Left Ventricular Summit; *LBBB* Left Bundle Branch; *RBBB* Right Bundle Branch; *PVC* Premature Ventricular Contractions.

### PVC Burden After 12-Month Follow-Up

Our results revealed a statistically significant reduction of 22.12% (95% CI 24.17, 20.06) in the mean Holter burden in the pediatric group (*p* < 0.001). Before the ablation procedure, the mean Holter burden in this group was 23.54 (SD = 8.32), which decreased to 1.42 (SD = 1.18) after the ablation procedure. Similarly, in the adult group, the mean Holter burden decreased from 26.37 (SD = 9.34) before ablation to 4.82 (SD = 7.89) after the procedure. This corresponds to a statistically significant reduction of 18.28% (95% CI: 23.89–19.21) (*p* = 0.001) (Table 4).

**Table 4:** Comparison between pre- and post-ablation PVC burden in both pediatric and adult groups after 12 months of follow-up

	Mean (SD)		Reduction (Mean, 95% CI)	*P*-value*
	Pre-ablation PVC burden (% of total cardiac beats)	Post-ablation PVC burden (% of total cardiac beats)		
Children (N = 61)	23.54 ± 8.32	1.42 ± 1.18	22.12 (24.17, 20.06)	< 0.001*
Adults (N = 101)	26.37 ± 9.34	4.82 ± 7.89	21.55 (23.89, 19.21)	0.001*

* Paired-samples T-test; significant p-value < 0.05

### Clinical Success

In the pediatric group, 58 out of 61 cases (95.08%) achieved clinical success, defined as a reduction of at least 80% in the Holter burden after ablation. Similarly, in the adult group, 75 (74.26%) out of 101 patients showed clinical success.

## Discussion

To our knowledge, this is the first study to compare the distribution of origin and clinical management of symptomatic high-burden idiopathic PVCs in the adult versus pediatric population. The main findings are that there are no significant differences in the prevalence of symptoms between both groups except syncope, which is higher in pediatrics. The incidence of RVOT-free walls is more common in pediatrics than in adults while septal RVOT and non-outflow tract PVCs are more common in adults. We found that there is no significant difference between both groups regarding the prevalence of PVC-induced myopathy. Finally, radiofrequency ablation outcomes and safety are similar in both groups.

### Clinical Presentation

In this study, the sense of palpitations was the most common symptom, on the other hand, syncope was significantly higher in children, although it was a rare condition. This variety of symptoms was explained by Lee A et al., they stated that symptoms are often not attributable to the PVC itself, but rather the sensation of the particularly strong heartbeat that occurs because of the prolonged ventricular filling time after that PVC, resulting in an enhanced stroke volume along the Frank-Starling curve and potentiated calcium release [13]. Regarding syncope, Zheng L et al. stated that some VVS (vasovagal syncope) patients experience symptomatic idiopathic PVCs due to elevated vagal tone, and they found that outflow tract PVCs, particularly RVOT, were the most frequently occurring arrhythmogenic foci in this patient cohort. [14] This is in accordance with the findings of the current study in the majority of patients who had syncope.

In the current study, only 2 pediatric patients had syncope due to ventricular arrhythmias, and those patients were diagnosed with PVC-induced myopathy. This was explained previously by Noda T. et al. In their study, benign forms of PVCs, such as those arising from the RVOT, may trigger polymorphic ventricular tachycardia or ventricular fibrillation [15]. A vulnerability to these tachyarrhythmias in response to a PVC or a combination remains unknown [16].

Regarding LV dysfunction, its prevalence was low in both groups (1.7% in the pediatric group and 5.8% in adults). This is similar to the previously published results of Chen et al. [17], who revealed that the prevalence of LV dysfunction in children was 1.2% (only 3 out of 249 children), while it was 8.6% in adults (13 out of 348), as stated by Hasdemir C et al. [4]

### PVC Origin

According to PVC morphology on the ECG and site of the successfully ablated PVCs, outflow tract PVCs were the most common origin, especially PVC-LBBB morphology in both groups. Although RVOT origin of PVC was the most common, it was noticed that the prevalence of non-outflow tract origin, especially LV summit and aortomitral continuity was more common in adults than in children. These findings are in concordance with previous studies, which revealed that PVC-LBBB, especially RVOT PVCs, are the most common forms of idiopathic PVCs in children [18, 19] and adults [20–22].

Interestingly, the current study revealed a higher prevalence of free wall RVOT in pediatric groups than in adults, while septal RVOT PVCs were more common in adults. This finding may be associated with the variance in RVOT volume, rotation, and volume of RV itself between children and adults. However, we could not discover any prior research mentioning this aspect, and we believe it should be considered in future larger trials with a larger sample size.

The low prevalence of non-outflow tract PVCs in children was also noticed by Beaufort-Krol et al. They noticed that the percentage of LV (PVCs-RBBB) in children with structurally normal hearts decreases spontaneously with age, while the percentage of premature beats originating from the RV (PVC-LBBB) does not change over time. The mean percentage of PVC-RBBB decreased from 16.3 ± 4.2% to 0.6 ± 1.4%, while the mean percentage of PVC-LBBB did not change (12.3 ± 21.4% vs. 11.7 ± 5.5%), with a p-value of 0.02 [18].

### Management

106 of 120 patients in the pediatric group received pharmacological therapy because of the refusal of their parents for RF ablation. Pharmacological therapy was effective only in 59 patients. 14 children were referred for RF ablation from the start due to the presence of syncope or LV dysfunction based on the recent guidelines [8]. They stated that ablation should be deferred in young and small children due to the risk of complications and the relatively larger size of the ablation lesion as compared to the child’s heart [8, 23, 24].

The effectiveness of AADs in this study was 55.6%, so 47 patients out of 106 (44%) were referred for ablation. These findings indicated the limited efficacy of AADs for lowering the PVC burden, in concordance with similar findings in previous studies [5, 24, 25].

### RF Ablation

In patients who underwent RF ablation, the acute success rate was high in both groups (≃ 90%) with rare complications and there was no significant statistical difference between adults and children regarding acute success and risk of complications. The high success rate of catheter ablation of idiopathic PVCs/VT has been reported with rare complications, particularly for the RVOT and fascicular types [11].

In a randomized study including patients with RVOT PVCs, ablation was superior to AADs for arrhythmia suppression with no differences in complications [26]. So, ablation is therefore recommended as the first-line therapy for RVOT and fascicular PVCs/VT in the recent guidelines. In children, there are still fewer studies than in adults, but they showed a high acute success rate with similar results and rare complications [3, 27].

### After One Year Follow Up

Catheter ablation in children revealed clinical success without the use of any AADs, even higher than that of adults. We explain that there is a lower prevalence of non-outflow PVC cases in children than in adults and a higher prevalence of RVOT PVCs in our study population. These findings are similar to the study done by Latchamsetty et al., who showed that the continued success at clinical follow-up with a mean of 1.9 years without the use of antiarrhythmic drugs was 71%. They also showed that the only significant predictor of continued success at clinical follow-up was a right ventricular outflow tract PVC location (*p* < 0.01), although this study included only adults, not children [11].

## Limitations

Our study was limited by the small number of included patients and a single medical center, which could affect the generalizability of the results. Additionally, short-term follow-up for just one-year could potentially obscure long-term complications and treatment outcomes.

## Conclusion

There was a difference between children and adults in the site of origin of idiopathic PVC and the prevalence of syncope associated with PVCs but no difference in the prevalence of PVC-induced myopathy, the treatment outcomes, or the safety of RF catheter ablation.

## Data Availability

No datasets were generated or analysed during the current study.

## Abbreviations

3D: Three Dimensional
AADs: Antiarrhythmic Drugs
AMC: Aorto- Mitral Continuity
BBB: Bundle Branch Block
CCBs: Calcium Channel Blockers
CI: Confidence Interval
^0^C: Celsius
ECG: Electrocardiography
EP: Electrophysiology
Fr: French
HIV: Human Immunodeficiency Virus
IQR: Interquartile Range
IRB: Institutional Review Board
LBBB: Left Bundle Branch Block
LCC: Left Coronary Cusp
LV: Left Ventricle
LVOT: Left Ventricular Outflow Tract
MRI: Magnetic Resonance Imaging
Ms: Milliseconds
N.Y: New York
PACES/HRS: The Pediatric and Congenital Electrophysiology Society /The Heart Rhythm Society
PVCs: Premature Ventricular Contractions
RBBB: Right Bundle Branch Block
RCC: Right Coronary Cusp
RF: Radiofrequency
RV: Right Ventricle
RVOT: Right Ventricular Outflow Tract
SD: Standard Deviation
USA: United States of America
VF: Ventricular Fibrillation
VT: Ventricular Tachycardia
WHO: World Health Organization
